# Lung Adenocarcinoma With Subintimal Spread Along the Pulmonary Artery: A Case Report

**DOI:** 10.7759/cureus.94429

**Published:** 2025-10-12

**Authors:** Kazuto Sugai, Shinji Kikuchi, Kojiro Nakaoka, Moriyuki Kiyoshima, Yuna Watanabe, Iijima Tatsuo

**Affiliations:** 1 Department of Thoracic Surgery, Ibaraki Prefectural Central Hospital, Kasama, JPN; 2 Department of Thoracic Surgery, University of Tsukuba, Tsukuba, JPN; 3 Department of Diagnostic Pathology, Ibaraki Prefectural Central Hospital, Kasama, JPN; 4 Department of Pathology, Ibaraki Prefectural Central Hospital, Kasama, JPN

**Keywords:** adenosquamous cell carcinoma, lung cancer, pulmonary artery invasion, subintimal invasion, vascular invasion

## Abstract

Pulmonary vascular invasion is a well-recognized feature of advanced lung cancer and is associated with poor prognosis. Vascular invasion usually refers to either microscopic invasion into small vessels or direct invasion of great vessels; however, longitudinal subintimal spread within the pulmonary artery has not been reported. We describe the case of a 77-year-old woman with a pulmonary nodule followed for two years, which enlarged to 23 mm with cavitation. A right upper lobectomy with mediastinal lymph node dissection was performed. Histopathology showed that the tumor was predominantly adenocarcinoma. In addition, longitudinal invasion was identified beneath the intima of the pulmonary artery, located between the lumen and elastic fibers without extension into the lumen or outside the vessel wall. Notably, in this subintimal invasive area, the central portion consisted of adenocarcinoma, whereas squamous differentiation was confined to the surrounding margins. The primary nodule measured 2.5 cm, but the total extent of subintimal invasion was 6 cm. The pathological stage was pT3N0, stage IIB. This case demonstrates a previously unreported pattern of pulmonary artery invasion by lung adenocarcinoma with characteristic marginal squamous differentiation, expanding the spectrum of vascular invasion in lung cancer.

## Introduction

Lung cancer remains one of the leading causes of cancer-related morbidity and mortality worldwide [1]. Various prognostic factors have been reported, and among them, pulmonary vascular invasion is recognized as one of the key pathological features associated with poor outcomes. Pulmonary vascular invasion, which is usually used to describe microscopic vascular invasion, is a well-recognized feature of advanced lung cancer and is associated with poor prognosis [2,3]. Microscopic vascular invasion, defined as the presence of tumor cells within endothelial-lined spaces, has repeatedly been identified as a significant adverse prognostic factor [4]. It is recommended for routine reporting in pathology protocols such as those of the College of American Pathologists (CAP) and is included in the WHO classification of thoracic tumors as a defining feature of invasion [4,5]. In contrast, macroscopic invasion of great vessels, including the pulmonary artery, is categorized as T4 disease in the IASLC/UICC TNM classification and is similarly regarded as vascular invasion and a high-risk feature [6]. Nevertheless, most descriptions of vascular invasion have focused on either microscopic intraluminal spread or direct transmural involvement of great vessels, and longitudinal spread beneath the intima of the pulmonary artery has not been common to date. Herein, we report a case of lung adenocarcinoma with longitudinal invasion along the peripheral pulmonary artery.

## Case presentation

**Figure 1:**
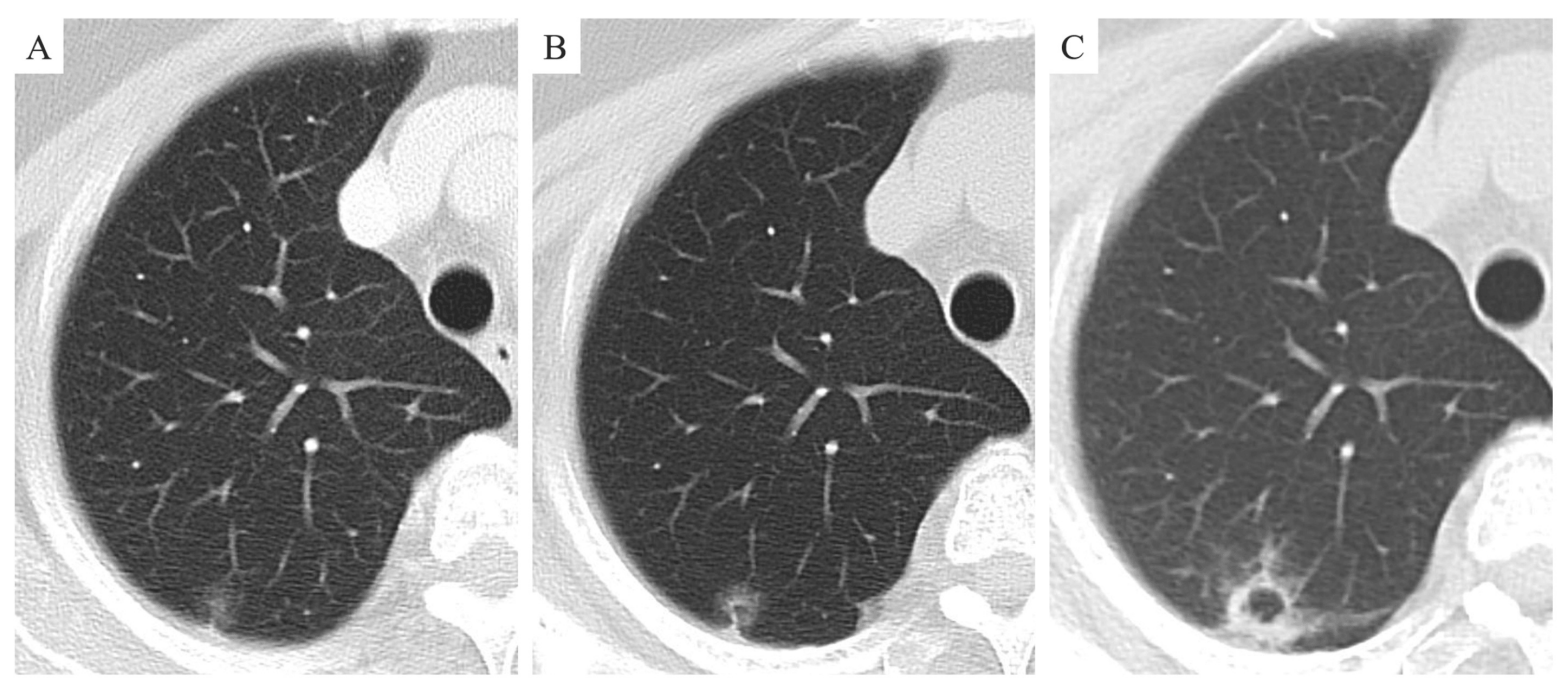
Computed tomography scans of the lung nodule over two years. Serial CT scans obtained at one-year intervals. A ground-glass nodule was initially detected (A), which gradually increased in size and density (A–C). By the end of follow-up, the nodule measured 23 mm and contained an internal cavity.

A 77-year-old woman, who had no respiratory symptoms and underwent a computed tomography (CT) scan to evaluate a coronary artery, was found to have an incidental pulmonary nodule that was followed up for two years. The nodule grew to 23 mm and contained a cavity (Figure 1).

Positron emission tomography revealed accumulated fluorodeoxyglucose with standardized uptake values of 4.9/6.3 (1 h/2 h, respectively). The nodule was suspected to be lung cancer of cT1cN0M0 cStageIA3 stage; therefore, a right-upper lobectomy with mediastinal lymph node resection was performed.

**Figure 2:**
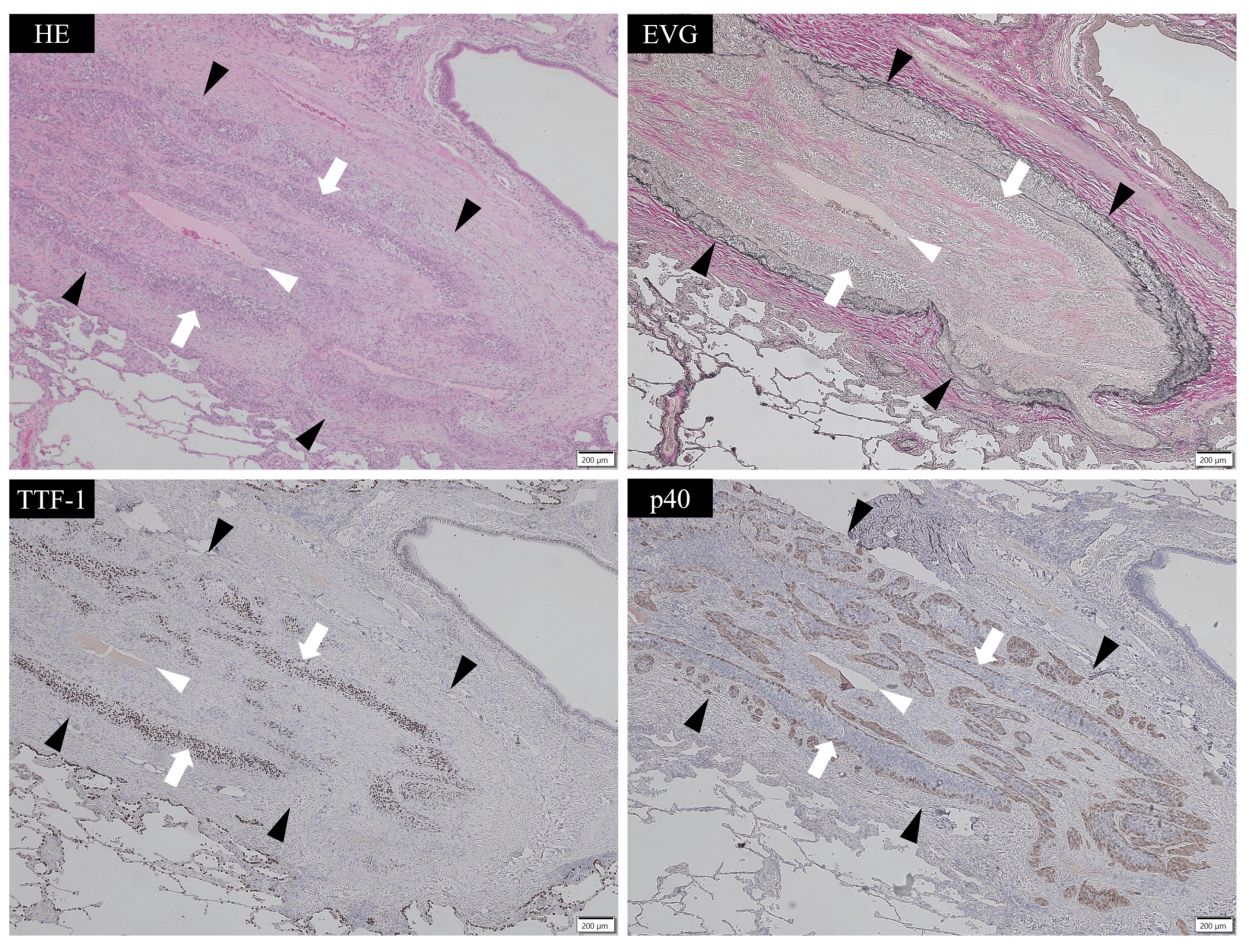
Histological findings of tumor invasion along the subintima of the pulmonary artery (low power). Hematoxylin and eosin (HE, upper left), Elastica van Gieson (EVG, upper right), thyroid transcription factor-1 (TTF-1, lower left), and p40 (lower right). White arrowheads indicate the pulmonary artery lumen, and black arrowheads indicate elastic fibers. Tumor cells (white arrows) were located between the intima and elastic fibers, but there was no invasion into the vascular lumen or outside of the vessel wall.

**Figure 3:**
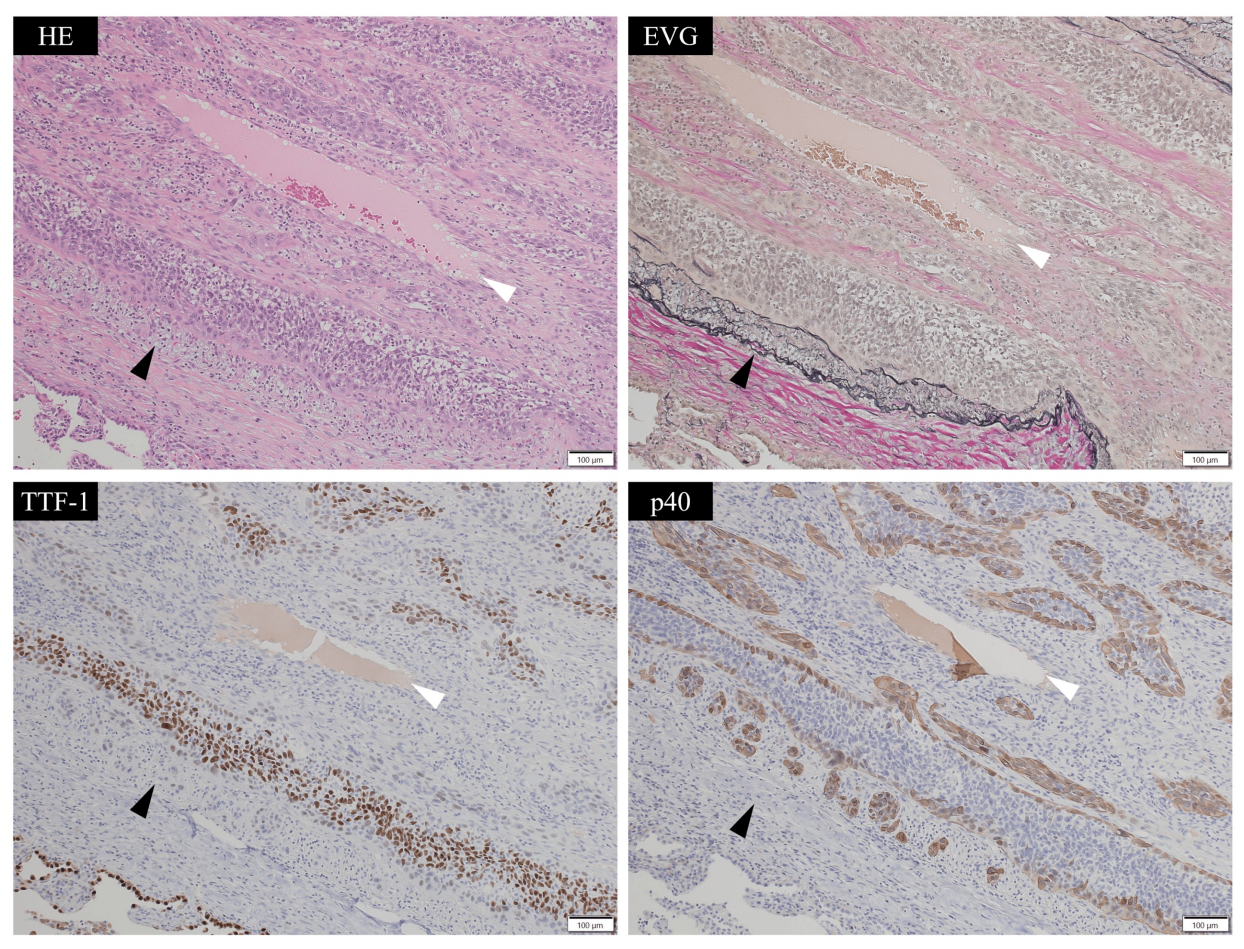
Histological findings of tumor invasion along the subintima of the pulmonary artery (medium power). Hematoxylin and eosin (HE, upper left), Elastica van Gieson (EVG, upper right), thyroid transcription factor-1 (TTF-1, lower left), and p40 (lower right). White arrowheads indicate the pulmonary artery lumen, and black arrowheads indicate elastic fibers. The tumor formed cellular islands; the central area consisted of TTF-1-positive adenocarcinoma cells, while the margins contained p40-positive and TTF-1-negative squamous cells.

Postoperative pathological examination revealed that the tumor mainly comprised irregular glandular duct structures within fibrous tissue. In addition, an unusual finding was observed in the pulmonary artery. Invasive tumor areas were located beneath the intima, between the vascular lumen and elastic fibers (the elastic layer that forms part of the arterial wall structure). Tumor cells were detected within the elastic fibers, but there was no invasion into the vascular lumen or outside of the vessel wall (Figures 2, 3).

**Figure 4:**
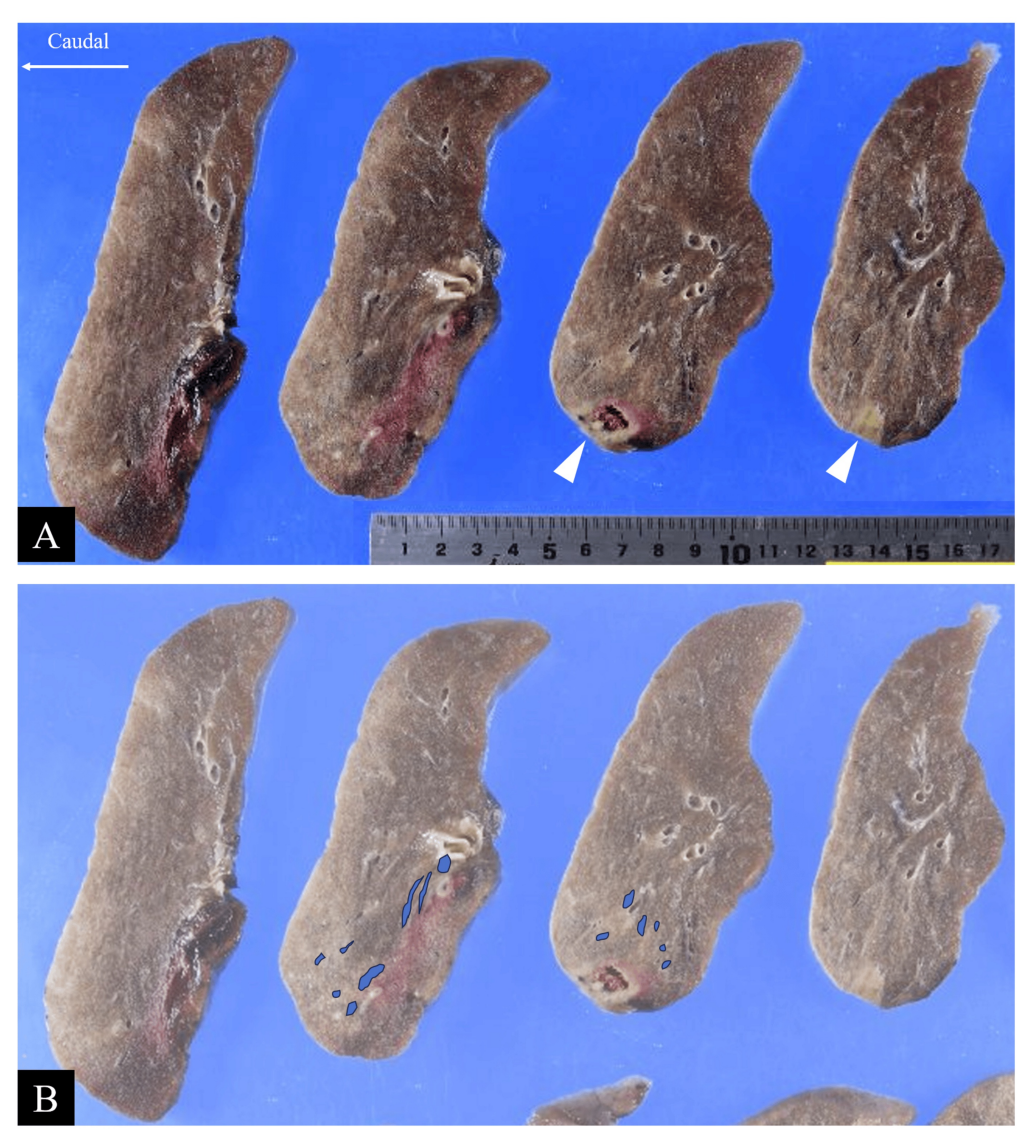
Macroscopic findings of the tumor. Horizontally sliced section of the resected right upper lobe. The tumor measured 2.5 cm in maximum diameter (white arrowheads). The upper row (A) shows the original specimen, while the lower row (B) highlights the extent of pulmonary artery invasion (blue areas). The total length of invasive extension was 6.0 cm.

The tumor also formed cellular islands; the central area consisted of thyroid transcription factor-1 (TTF-1)-positive adenocarcinoma cells, while the margins contained p40-positive and TTF-1-negative squamous cells (Figures 2, 3). Macroscopically, the invaded artery was found to run along the bronchus, but no tumor cells were identified within the bronchial wall (Figure 4).

Overall, the tumor was diagnosed as adenocarcinoma with focal squamous differentiation (<10%). The adenocarcinoma subtypes were lepidic (30%), acinar (50%), and solid (20%). The primary nodule measured 2.5 cm at its greatest dimension, but the total length of the invasion, including the subintimal extension along the pulmonary artery, was 6 cm (Figure 4). Consequently, the pathological stage was pT3N0, stage IIB. Genetic analysis revealed mesenchymal-epithelial transition exon 14 skipping. The postoperative course was uneventful. However, disease relapse was detected at eight months after surgery, involving the bronchial stump, lymph node metastasis, pleural dissemination, and multiple bone metastases. The patient was treated with a MET tyrosine kinase inhibitor, followed by chemoimmunotherapy, and remains alive at 28 months postoperatively.

## Discussion

This case is unique in two respects: the longitudinal subintimal invasion (tumor extension along the layer just beneath the vascular intima) of the pulmonary artery and the histology demonstrating adenocarcinoma centrally with squamous differentiation at the invasive margins. Vascular invasion typically refers to microscopic tumor spread into small vessels, which is widely recognized as a poor prognostic factor [2,3]. It is recommended for routine reporting in pathology standards such as the CAP protocol, and the WHO classification incorporates vascular invasion within its definition of invasion [4,5]. According to the CAP, vascular invasion is defined as the presence of tumor cells within endothelial-lined vascular spaces, including veins and arteries [4]. By this definition, the present case cannot be classified as microscopic vascular invasion, because no tumor was present within the vascular lumen. In other words, vascular invasion can also refer to the invasion of the great vessels. The IASLC/UICC TNM classification (8th edition) defines direct macroscopic or histologic invasion of great vessels, such as the pulmonary artery, as T4 disease [6]. However, in the present case, the tumor invaded the peripheral pulmonary artery without evidence of invasion from outside. Thus, the subintimal longitudinal invasion observed here does not meet the conventional definition of great vessel invasion either.

With regard to longitudinal tumor extension, invasion along the bronchial wall is relatively common in lung cancer [7,8]. In such cases, bronchial wall invasion is usually identified as wall thickening on CT. In contrast, in the present case, no tumor was observed in the bronchial wall or broncho-vascular band, and no arterial abnormalities were detected preoperatively. Among lung tumors with marked affinity for the pulmonary artery, pulmonary angiosarcoma has been reported to extend longitudinally within the lumen [9], but this entity differs both pathologically and clinically. A somewhat related case of adenocarcinoma was reported by Goto et al., in which the tumor invaded the pulmonary artery, formed an intraluminal polypoid mass, and replaced the endothelial lining with adenocarcinoma [10]. However, the subintimal longitudinal invasion without luminal protrusion observed in the present case has not, to our knowledge, been previously described. Because this type of invasion is not detectable on preoperative imaging, and intraoperative frozen section analysis is usually not performed, there is a potential risk of positive surgical margins. Therefore, subintimal invasion of the pulmonary artery may lead to postoperative local recurrence. However, as similar cases have not been reported to date, it is difficult to assess their prognostic significance, and careful postoperative follow-up is warranted.

Adenosquamous carcinoma accounts for approximately 0.4-4.0% of all lung cancers and comprises both adenocarcinoma and squamous carcinoma components [11]. It is characterized by distinct cellular populations, sometimes separated and sometimes intermixed. Smaller tumors often show clear separation between components, whereas larger tumors tend to display mixed features, suggesting collision-type development [12]. In our case, adenocarcinoma and squamous cells were intermixed, but the distribution was orderly and aligned, not resembling a simple collision of two cancers. Two alternative hypotheses for the pathogenesis of adenosquamous carcinoma, differentiation from multipotent stem cells [13] or transdifferentiation from one histology to the other [14], do not fully explain the concentric arrangement seen here. The presence of squamous differentiation surrounding an adenocarcinoma component has not been previously documented.

## Conclusions

This case demonstrates a previously unreported pattern of lung adenocarcinoma with longitudinal subintimal invasion of the pulmonary artery and characteristic marginal squamous differentiation. Unlike conventional microscopic vascular invasion or direct great vessel invasion, this growth occurred beneath the intima without intraluminal protrusion or transmural extension, highlighting the importance of thorough histopathological assessment beyond imaging findings. Because of its rarity, the prognostic significance remains uncertain; however, awareness of this distinctive mode of vascular involvement is clinically relevant, as it may carry implications for local recurrence. Recognition and reporting of similar cases will be essential for broadening our understanding of vascular invasion patterns and tumor progression in lung cancer.
